# CMV Infection and CMV-Specific Immune Reconstitution Following Haploidentical Stem Cell Transplantation: An Update

**DOI:** 10.3389/fimmu.2021.732826

**Published:** 2021-10-28

**Authors:** Xiao-Hua Luo, Yan Zhu, Yu-Ting Chen, Li-Ping Shui, Lin Liu

**Affiliations:** ^1^ Department of Hematology, The First Affiliated Hospital of Chongqing Medical University, Chongqing, China; ^2^ Department of Hematology, Southwest Hospital, Third Military Medical University (Army Medical University), Chongqing, China

**Keywords:** cytomegalovirus, infection, immune reconstitution, haploidentical, stem cell transplantation

## Abstract

Haploidentical stem cell transplantation (haploSCT) has advanced to a common procedure for treating patients with hematological malignancies and immunodeficiency diseases. However, cure is seriously hampered by cytomegalovirus (CMV) infections and delayed immune reconstitution for the majority of haploidentical transplant recipients compared to HLA-matched stem cell transplantation. Three major approaches, including *in vivo* T-cell depletion (TCD) using antithymocyte globulin for haploSCT (*in vivo* TCD-haploSCT), *ex vivo* TCD using CD34 + positive selection for haploSCT (*ex vivo* TCD-haploSCT), and T-cell replete haploSCT using posttransplant cyclophosphamide (PTCy-haploSCT), are currently used worldwide. We provide an update on CMV infection and CMV-specific immune recovery in this fast-evolving field. The progress made in cellular immunotherapy of CMV infection after haploSCT is also addressed. Groundwork has been prepared for the creation of personalized avenues to enhance immune reconstitution and decrease the incidence of CMV infection after haploSCT.

## Introduction

**Figure 1:**
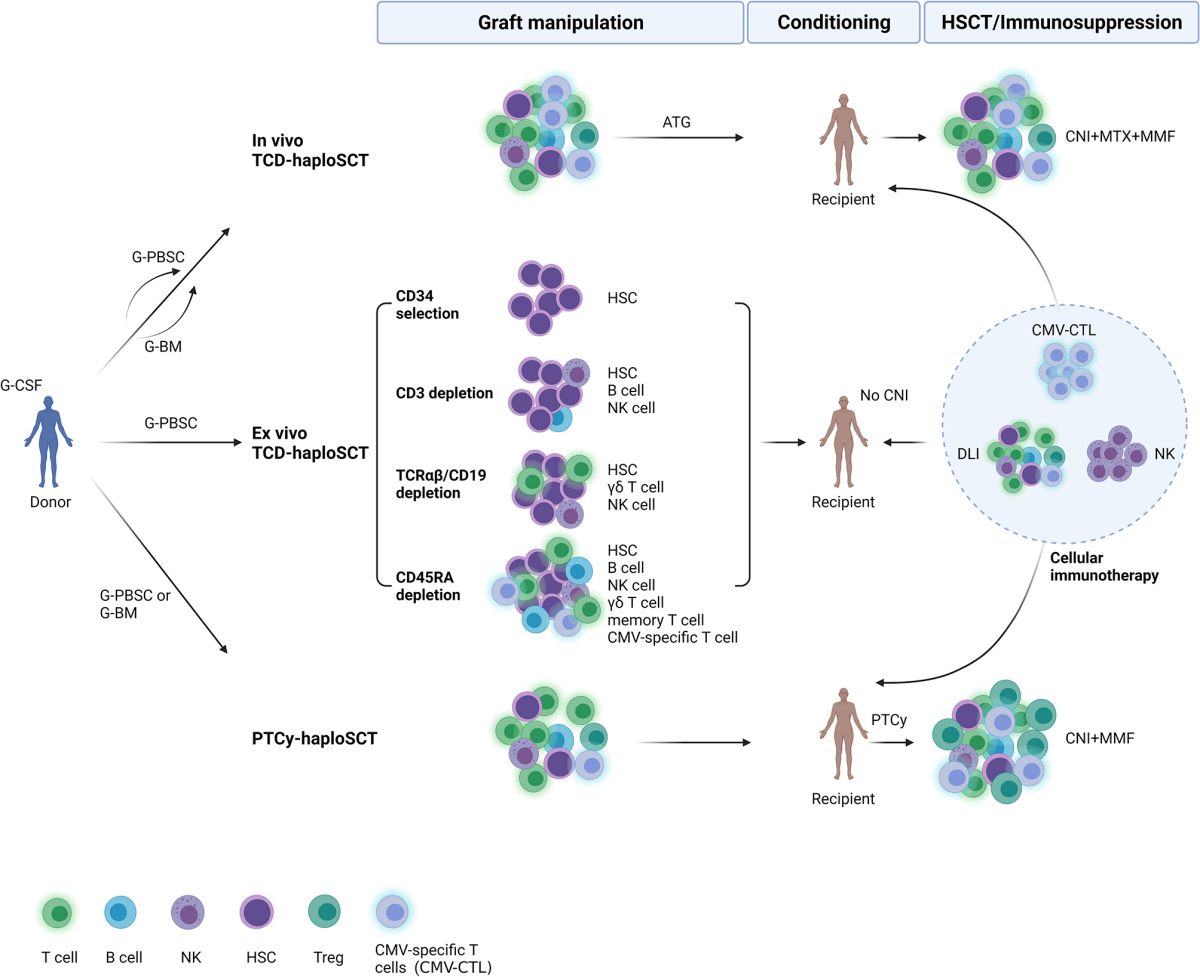
Overview of immune reconstitution to cytomegalovirus and cellular immunotherapy after three major approaches of haploidentical stem cell transplantation (haploSCT). In vivo TCD-haploSCT, in vivo T-cell depletion (TCD) using antithymocyte globulin for haploSCT; Ex vivo TCD-haploSCT, ex vivo TCD using CD34 + positive selection for haploSCT; PTCy-haploSCT, T-cell replete haploSCT using posttransplant cyclophosphamide. G-CSF, granulocyte-colony stimulating factor; G-PBSC, G-CSF primed peripheral blood stem cells; G-BM, G-CSF primed bone marrow; HSC, hematopoietic stem cell; CMV, cytomegalovirus; CNI, calcineurin inhibitors; MTX, methotrexate; MMF, mycophenolate mofetil; DLI, donor lymphocyte infusion; NK cell, natural killer cell; Treg, regulatory T cell; HSCT, hematopoietic stem cell transplantation. Created with BioRender (https://biorender.com/).

HLA-haploidentical stem cell transplantation (haploSCT) has spread rapidly worldwide in recent years. HLA-haploidentical donors sharing a single HLA haplotype with transplant recipients are almost always available, so haploSCT can be performed for patients who are lacking HLA-matched donors and/or are urgently needing transplantation. The major approaches for T-cell depletion are *in vivo* T-cell depletion using antithymocyte globulin (ATG) (*in vivo* TCD-haploSCT), *ex vivo* T-cell depletion (TCD) using CD34 + positive selection (*ex vivo* TCD-haploSCT), and T-cell replete haploSCT using posttransplant cyclophosphamide (PTCy-haploSCT). Compared with HLA-identical sibling transplantation, patients undergoing haploSCT usually receive more intensive immunosuppressors to guarantee engraftment and later prevent graft-versus-host disease (GVHD). Therefore, these patients always have impaired immune reconstitution after transplantation and a high incidence of CMV infection and CMV disease (**Figure 1**). As the use of haploidentical transplantation has increased substantially, we summarize current data on CMV infection and its immune reconstitution after haploSCT during the last decade.

## Incidence of Cytomegalovirus Infection After haploSCT

### *In Vivo* TCD-haploSCT (Anti-Thymocytic Globulin/ATG-Based)

Using the Beijing protocol at Peking University (1–7), there was a high incidence of CMV reactivation early after haploSCT (59.5-66%), whereas the rate of CMV disease was actually low (2.92-17%). CMV DNAemia was initially detected after a median of 35 days with a mean duration of positivity of 15 days (5, 6). Most (91.2%) cases of CMV gastroenteritis developed within 100 days, whereas most (90.3%) cases of CMV retinitis were late onset with the cumulative incidence of CMV retinitis at 2.3% one year (a median onset of 167 days) after haploSCT (6, 7). Einat Shmueli et al. from Israel designed a conditioning protocol for haploSCT including fludarabine, thiotepa, anti-thymocytic globulin, and total body irradiation (8). After receiving preemptive therapy, the incidence of CMV infection was 66.7% in haploSCT, and 11.6% of haploSCT transplant recipients with CMV reactivation developed CMV disease. Importantly, drug-resistance mutations and clinically suspected resistance were discovered only in haploSCT recipients (8), favoring prophylactic over preemptive treatment in high-risk patients and highlighting the need for better anti-CMV drugs.

It remains unclear whether primary disease affects CMV infection after haploSCT. Lan‐Ping Xu et al. from Peking University conducted studies to confirm the feasibility of haploidentical transplantation in patients with severe aplastic anemia (SAA) as salvage therapy (9–12). CMV viremia occurred in 51.7~84.00% of SAA patients. However, no difference in the rates of early CMV disease between haploidentical patients and matched related patients was found (9, 10). Consistently, several centers in China obtained similar results for SAA patients (13–15). The haploSCT cohorts with AML, MDS, or Ph+ ALL, including haplo-cord-HSCT, had higher CMV viremia than the HLA-matched HSCT cohorts (16–19), but the incidence of CMV disease was not significantly different between the two groups. Even in pediatric patients with MDS or patients with relapsed/refractory acute lymphoblastic leukemia after CAR-T therapy who underwent haploSCT, the incidence of CMV reactivation/infection was less than 60%, and very few patients developed CMV disease (20, 21).

Using a similar protocol, several transplant centers have reported promising results for unmanipulated haploidentical peripheral blood stem cell transplantation (PBSCT) (22, 23) or cotransplantation of unrelated cord blood (UCB) (24–26) or mesenchymal stem cells (MSCs) (27, 28). The 1-year cumulative incidence of CMV DNAemia in patients with hematologic malignancies was 23.5-41.7% in the matched sibling donor (MSD)-SCT group versus 62.1-81.0% in the haploSCT group with peripheral blood stem cells (PBSCs) (29, 30). The median time to the onset of CMV DNAemia in the haploSCT group was 33 days (range, 10–159 days) with the 1-year cumulative incidence of CMV disease at 7.9% (95% CI, 3.6–14.3%) (29). In addition, a total of 19.4%-92% of these patients experienced CMV reactivation after combination of haploSCT with UCB or MSCs (24–28). There was no statistical significance in the incidence of CMV viremia in terms of haplo-cord SCT *vs* HLA-matched donor SCT (MD-SCT) or haplo-cord SCT *vs* haploSCT (24–26).

As the use of ATG as a regimen for *in vivo* TCD and immunosuppressants is limited by impaired immune restoration and a high risk of severe infections, researchers are working on their impact after haploSCT. Peking University performed a study comparing 6 mg/kg ATG versus 10 mg/kg ATG in patients who underwent haploSCT (31). The 1-year cumulative incidence of CMV reactivation was similar between the ATG-6 and ATG-10 groups[(75.0% (66.8–83.2%) *vs* 78.6% (75.2–82.0%)]. Another multicenter study investigated the impact of 7.5 mg/kg and 10.0 mg/kg rabbit ATG on GVHD and virus reactivation after haploSCT (32). The 1-year incidence of CMV DNAemia was higher in the 10.0 mg/kg group [83.4% (77.5-87.9)] than in the 7.5 mg/kg group [73.4% (67.2-79.4)], whereas the 2-year incidence of CMV-associated diseases was also higher in the 10.0 mg/kg group [5.9% (3.2–9.7%)] than in the 7.5 mg/kg group [1.5% (0.4–4.0%)]. Yu Wang et al. recently extended follow-up from this original trial (33). They found that patients undergoing haploSCT benefit from 7.5 mg/kg ATG compared to 10.0 mg/kg ATG based on a balance between GVHD and infection control. The data supports ATG (7.5 mg/kg) is potentially the standard regimen in this platform. Researchers from Japan and the Republic of Korea later performed haploSCT using low-dose thymoglobulin at 5 mg/kg (34, 35). CMV reactivated in 41.67% and 72.7% of patients, but CMV disease developed in 0 and 19.4% of patients, respectively. A recent report from the Republic of Korea indicated that the cumulative incidence of CMV DNAemia at 3 years was 45.7% (30.7-59.4) for ATG (5-10 mg/kg)-based haploSCT (36). Moreover, a short-term tacrolimus regimen for the prophylaxis of GVHD in haploSCT did not increase the incidence of CMV infection compared with the Cyclosporine A regimen (39.5% *vs*. 37.5%, p = 0.783) (37).

### *Ex Vivo* TCD-haploSCT

CD34+ selection was initially used as a method for TCD, but it resulted in delayed immune reconstitution and a high incidence of opportunistic infections and nonrelapse mortality. The *ex vivo* TCD techniques have developed from CD34+ selection, CD3+ cell depletion, and αβ+/CD19+ cell depletion to recent CD45RA+ depletion. Compared with CD34+ cell selection, after CD3+ cell depletion, the graft has more natural killer (NK) cells, monocytes, and other immunomodulating cells with better outcomes. Sameh Gaballa et al. retrospectively compared data on patients undergoing a two-step (a fixed T cell infusion followed 2 days later by cyclophosphamide, and then a CD34-selected stem cell product infused) haploidentical or matched related PBSCT for high-risk hematological malignancies and aplastic anemia (38). Compared with the matched related PBSCT group (matched related, 19%), the 100-day cumulative incidence of CMV viremia was higher in the haploidentical group (haploidentical, 67%). The median time to develop CMV reactivation was 26 days in the haploSCT group and 36 days in the matched related PBSCT group.

The cumulative incidence of CMV DNAemia in patients with acute leukemia was 73.5-81% after *ex vivo* αβ T cell-depleted haploSCT (39, 40). No patient developed CMV disease or died (39). A more recent study explored the role of interim-foscarnet prophylaxis in preventing CMV infection after *ex vivo* αβ T cell-depleted haploSCT in children between May 2012 and May 2018 (41). Forty (50.8%) of 81 patients developed CMV reactivation at a median of 41.3 days (range, 13–132) after haploSCT. The median duration of CMV reactivation was 28.5 days (range, 1–179), and the peak PCR level was 3.82 log copies/mL (range, 2.85–6.03) (41). In nonmalignant disease, ganciclovir/foscarnet significantly decreased CMV reactivation incidence (43.7% *vs*. 78.3%), whereas the prophylaxis strategy had no significant impact in patients with hematological malignancies. No significant difference was found in the rate of CMV disease according to prophylaxis method. It suggests that this intensified antiviral strategy may be necessary for αβ T cell-depleted haploSCT patients with nonmalignant disease who require higher doses of ATG.

Through TCR α+β+/CD19+ cell-depleted haploSCT, it is feasible to transfer to the transplant recipient both donor hematopoietic stem cells (HSCs) and hematopoietic progenitors as well as NK and γδ T cells, which could protect against leukemia and life-threatening infections, including posttransplant lymphoproliferative disease (PTLD). A total of 7.27-75% of patients undergoing TCR α+β+/CD19+ cell depleted HSCT experienced CMV reactivation (42–46). Most patients experienced CMV viremia during the first month after haploSCT (days +1 to +24) (45). In a report including three sickle cell disease and 11 thalassemia patients, Gaziev J et al. stated that viral reactivation occurred in the vast majority of patients after TCR α+β+/CD19+ cell–depleted haploSCT, with CMV reactivation in 64%, although no cases of CMV were noted (47).

After removal of potentially alloreactive CD45RA+ cell depletion, memory T cells, including virus-specific T cells left in grafts, could shorten viremia and reduce GVHD (48). B M Triplett et al. reported data from 17 patients with poor-prognosis hematologic malignancy who underwent haploSCT with CD45RA-depleted grafts after a reduced intensity conditioning regimen without TBI or serotherapy (49). Three patients of 17 received anti-CMV treatment after CMV reactivation. None of the patients experienced CMV disease, and all of them cleared CMV viremia without donor lymphocyte infusion (DLI). Early T-cell reconstitution was directly linked to the CD45RA-depleted graft content. This group then compared 41 patients receiving CD3‐depleted (CD3dep recipients) grafts with 26 receiving CD45RA‐depleted grafts (CD45dep recipients) after haploSCT (50). CD3dep recipients were more likely to develop CMV reactivation—23 (56%) *vs* 5 (19%). All CD3dep recipients with CMV received treatment, and eight (36%) were also infused with donor lymphocytes for CMV, whereas CMV treatment was needed for only three of the five CD45RAdep recipients. Although three CD3dep recipients died with active CMV viremia, CMV was not detected in CD45RAdep recipients at the time of death. It seems that CD45RA-depleted haploSCT confers enhanced T-cell recovery and reduced infection without increase in severe GVHD among these *ex vivo* TCD methods.

### PTCy-haploSCT

PTCy is a method of *in vivo* T cell depletion that mainly acts on alloreactive T cells after haploSCT. CMV reactivation was noticed in 42%-69.2% of patients who underwent PTCy-haploSCT (51–58). A total of 2.8%-4.5% of patients experienced CMV-associated disease (51, 52). CMV reactivation occurred at a median time of 35-39 days (51, 52, 57). The median time to first episode of CMV DNAemia was 33 days (range, −7 to 123 days) after haploSCT (58). Moreover, the CMV DNA peak load was remarkably higher in haploSCT recipients, but the mortality by days 180 and 365 did not differ among comparison groups (55). García-Cadenas Irene et al. studied the impact of HLA donor matching on infection in patients receiving PTCy-based alloSCT (59). They found that haploSCT recipients had a higher incidence of CMV infection/reactivation at 18 months than other transplant modalities [(61% (95% CI: 41–74%) *vs*. 44% (95% CI: 31–54%)], whereas lethal infections were uncommon across all these groups. In their study, severe infections were common in transplant patients using PTCy. A more recent CIBMTR analysis reported (51) that PTCy increased the risk of CMV infection among CMV-seropositive recipients in both haploSCT and matched sibling donor HSCT compared with calcineurin inhibitor–based sibling donor transplantation, suggesting intensive CMV prevention strategies in all receiving PTCy. This is supported by the fact that an intensified method to prevent CMV reactivation correlated with a lower incidence of CMV reactivation (67% intensified group versus 81% traditional group) and less CMV disease (0% hybrid/intermediate dose versus 8% traditional dose) without increased toxicity after PTCy-haploSCT compared with a traditional antiviral prophylaxis regimen (60).

Primary disease and conditioning regimen could also impact CMV infection after PTCy-haploSCT. CMV reactivation post engraftment was noted in 43.7% and 62% of transplant recipients with primary immune deficiency disorders (PIDs) (61) and relapsed/refractory SAA (62) undergoing PTCy-haploSCT, respectively. R V Raj et al. then investigated the effect of conditioning intensity on the incidence of viral infection after PTCy-haploSCT (63). Their study found that challenging viral infections after haploSCT cause significant morbidity in this patient population. It appears that the incidence of viral complications is higher following myeloablative doses of busulfan-containing conditioning regimens (63). Emmanuel Katsanis et al. recently performed a single center phase I study substituting day +4 PTCy with bendamustine (PT‐BEN) following myeloablative conditioning and T‐cell replete haploidentical bone marrow transplantation (64). CMV reactivation was notably less common in trial patients receiving PTCy/BEN, with one out of eight at-risk (seropositive recipient and/or seropositive donor) of experiencing CMV reactivation, whereas 71.4% of the at‐risk PTCy patients reactivated CMV.

Compared with bone marrow (BM) as a graft source, PBSCs could yield higher CD34+ cell counts but were possibly accompanied by increased GVHD; however, no difference in GVHD was observed in haploSCT (65). A total of 46-68% of patients with PTCy-haploSCT and PBSC grafts had posttransplant CMV viremia (65–69). The median time to viremia was 24 days (range: 3–68). CMV disease occurred in 17-28.8% of patients with CMV viremia (65, 68). Sirolimus with micophenolate mofetil (MMF) has recently been regarded as an alternative to calcineurin inhibitor-containing approaches, as this combination has a decreased risk of acute renal failure, decreased incidence of CMV reactivation, and better regulatory T cell reconstitution. Some groups have introduced PTCy plus sirolimus and MMF (PT-CY-Sir-MMF) as GVHD prophylaxis in allo-HSCT, regardless of donor type (70, 71). CMV DNAemia occurred in 52-63% of patients after haploSCT. The cumulative incidence of CMV DNAemia in patients who received pre-emptive antiviral therapy at one year was 39% (95% CI, 31–47%), and the 1-year cumulative incidence of CMV disease was 2.6% (95% CI, 0.09–5%) (70).

### ATG+ PTCy-haploSCT

As ATG is usually used to reduce the risk of graft rejection and GVHD, it is assumed that ATG combined with PTCy in T-cell replete-haploSCT would minimize GVHD risk but not impact engraftment and risk of relapse. Princes Margaret Cancer Centre from Canada established unmanipulated haploidentical PBSC transplantation following RIC with ATG (total 4.5 mg/kg), PTCy (cyclophosphamide 50 mg/kg/day i.v. on days +3 and +4), and cyclosporine as a GVHD prevention strategy (72–74). CMV reactivation occurred in 74% of cases with CMV disease in 11.5% of cases (72). Cheng‐Hsien Lin et al. retrospectively compared the cumulative incidence of CMV DNAemia, two‐year OS, and leukemia‐free survival rates in acute leukemia patients with MSD, matched unrelated donor (MUD), and haploidentical donor allografts (ATG: 2 mg·kg-1 day-1, from day -3 to day -2; PTCy) (75). The cumulative incidences of CMV DNAemia at day 180 in the haploidentical groups were 85.7%, which were higher than those in the MSD and MUD allo‐HSCT groups. For the haploidentical groups, CMV DNAemia was detected at a median time of 29 days.

Yu Wang et al. from Peking University initiated a prospective study in patients with a standard-dose ATG/granulocyte colony-stimulating factor (G-CSF)-based regimen (ATG-PTCy) followed by low-dose PTCy (14.5 mg/kg on days 3 and 4) for haploSCT (76, 77). The 100-day cumulative incidence of CMV reactivation in the ATG-PTCy cohort was markedly higher than that in the ATG cohort (74% *vs* 30%), with a comparable incidence of CMV disease between the two cohorts (8% *vs* 8%) (77), indicating that dual T cell depletion with PTCy and ATG may bring about a higher incidence of CMV reactivation.

### Comparison Among These Approaches

Published data have been inconsistent on the incidence of CMV reactivation and CMV disease after haploSCT (**Table 1**). It indicates that haploSCT carries a substantially higher risk for CMV infection compared with HLA‐matched related or unrelated allo‐HSCTs, but this seemed not to impact overall and non‐relapse mortality. Hence, some data suggest the use of prophylactic anti-CMV antivirals when PTCy is used because a higher incidence of CMV reactivation was associated with the use of PTCy (51, 60). Surprisingly, a systematic review and meta-analysis of studies on haploSCT in idiopathic AA suggested that the addition of PTCy correlated with a lower risk of CMV viremia (10.4%) to a larger extent than MTX-containing (55.7%) and other (38.6%) regimens (79). The opposite results can be partly explained by the absence of an approved threshold of viral load to initiate anti-CMV treatment, considering the different transplant centers. The heterogeneous CMV serological status in the donor/recipient on account of geographical and ethnological characteristics is another possible explanation because the CMV seroprevalence is usually much higher (>=90%) in adult populations of China than in Europe and the USA (80–84). This issue could be better investigated in a future clinical trial.

**Table 1:** Selected reports on CMV infection after haploidentical stem cell transplantation.

Group	Year	Country	haploSCT Sample size	Primary Disease (n)	Stem cell source (n)	Graft manipulation	Dose of ATG	Conditioning (n)	GVHD prophylaxis	Assays measuring CMV DNAemia	Cutoff values for CMV reactivation or reactivation needing PET	CMV reactivation	CMV disease	Clinical outcome/Comments	Reference
Y Wang et al.	2013	China	756	AML (321); ALL (299); CML (136)	BM+PBSC	*in vivo* TCD-haploSCT	r-ATG 2.5 mg/kg×4d	Modified BUCY	CsA+MMF+short-term MTX	Real-time PCR or with a CMV pp65 antigenemia test	NR	100-day 64%	4%	2-year relapse (18%); 3-year OS (67%), LFS (63%), NRM (18%). More CMV-seropositive patients became antigenemia-positive than CMV-seronegative patients.	(4)
Y Chen et al.	2016	China	248	AL (201); CML (32); Others (15)	BM+PBSC	*in vivo* TCD-haploSCT	r-ATG 2.5 or 1.5mg/kg×4d	Modified BUCY (241); TBI+CY+Me-CCNU (7)	CsA+MMF+short-term MTX	Real-time PCR (RT-PCR)	A viral load of >500 copies/ml for two consecutive readings 5 days apart	59.50%	6.85%	CMV DNAemia was found to be a poor prognostic factor in terms of NRM and OS. HBsAg seropositivity was associated with an increased risk of cytomegalovirus DNAemia.	(5)
CH Yan et al.	2020	China	1466	AML (801); ALL (490); MDS (175)	BM+PBSC	*in vivo* TCD-haploSCT	r-ATG 2.5 mg/kg×4d	Modified BUCY (1416); TBI+CY+Me-CCNU (50)	CsA+MMF+short-term MTX	Automated, real-time, quantitative PCR assay	A detection threshold of >1000 copies/ml was defined as positive	64.80%	1-year CMVR 2.3%	CMVR was a rare complication after haploidentical HSCT but that the risk was greater in patients with multiple risk factors.	(6)
XY Meng el al.	2020	China	3862	AML (36); ALL (51); MDS (14); CML (4); SAA (2); Others (6)	BM+PBSC	*in vivo* TCD-haploSCT	r-ATG 2.5 mg/kg×4d	Modified BUCY or TBI+CY+Me-CCNU BUCY (SAA)	CsA+MMF+short-term MTX	Real-time PCR	A limit of detection of 509 IU/mL	NR	2.92%	1 year NRM 34.9% in patients with CMV diseases	(7)
LP Xu et al.	2016	China	101	SAA	BM+PBSC (100); BM (1)	*in vivo* TCD-haploSCT	r-ATG 2.5 mg/kg×4d	BUCY	CsA+MMF+short-term MTX	NR	NR	68.30%	1%	3-year OS (89.0%); FFS (86.8%)	(9)
LP Xu et al.	2017	China	89	SAA (69); VSAA (20)	BM+PBSC (78); BM (9); PBSC (2)	*in vivo* TCD-haploSCT	r-ATG 2.5 mg/kg×4d	BUCY	CsA+MMF+short-term MTX	NR	NR	51.70%	1.12%	3-year OS (86.1 ± 3.7%); FFS (85.0 ± 3.9%)	(10)
LP Xu et al.	2018	China	51	SAA	BM+PBSC	*in vivo* TCD-haploSCT	r-ATG 2.5 mg/kg×4d	BUCY	CsA+MMF+short-term MTX	NR	NR	84.00 ± 0.29%	1.96%	1- and 3-year OS 83.5 ± 5.4% (the probabilities of FFS were equal to the OS)	(11)
LP Xu et al.	2017	China	52 pediatric patients	SAA (32); VSAA (20)	BM+PBSC	*in vivo* TCD-haploSCT	r-ATG 2.5 mg/kg×4d	BUCY	CsA+MMF+short-term MTX	NR	NR	69.20%	NR	3-year OS (84.5 ± 5.0%); FFS (82.7 ± 5.2%)	(12)
Y Lu et al.	2018	China	41	SAA	BM+PBSC	*in vivo* TCD-haploSCT	r-ATG 7.5 mg/kg (total dose) ATG-F 20mg/kg (total dose)	BUCY	Tacro+MMF+short-term MTX	PCR	Higher than 500 copies/mL	65.90%	4.88%	3-year OS (80.3 ± 5.1%); FFS (76.4 ± 5.1%); GFFS (79.0 ± 8.6%)	(13)
L Liu et al.	2020	China	146	SAA (75); VSAA (71); SAA with PNH clone (15)	BM (15); PBSC (4); BM + PBSC (127)	*in vivo* TCD-haploSCT	r-ATG 2.5 mg/kg×4d	BUCY	CsA+MMF+short-term MTX	Real-time PCR	NR	42.47%	2.05%	4-year OS (81.4 ± 3.3%); GFFS (69.2 ± 3.9%)	(24)
Z Liu et al.	2017	China	44	SAA (31); VSAA (13)	BM+PBSC+MSCs	*in vivo* TCD-haploSCT	r-ATG 3.125 mg/kg×4d	BUCY	CsA+MMF+short-term MTX	NR	NR	65.90%	0	2-year OS 77.3%	(27)
Z Wang et al.	2014	China	17 children and adolescents	SAA (11); VSAA (5); 2nd HSCT (1)	BM+PBSC+MSC	*in vivo* TCD-haploSCT	r-ATG 5mg/kg×4d (-4 to -1); ALG 20mg/kg/day d-4 to -1	Flu+BUCY	CsA+MMF+short-term MTX+basiliximab	Real-time PCR	NR	82.30%	0	1-year OS 71.60 ± 17.00%	(14)
L Gao et al.	2014	China	26	SAA (16); VSAA (10)	BM+PBSC	*in vivo* TCD-haploSCT	r-ATG 2.5 mg/kg×4d	Flu+CY	CsA+MMF+short-term MTX	PCR	NR	23.08%	3.85%	TRM 3.8% (100-day), 11.5% (1-year), 15.4% (2-year); OS 84.6% (follow-up of 1313.2 days)	(15)
Y Lu et al.	2021	China	377	AML	BM+PBSC	*in vivo* TCD-haploSCT	r-ATG 7.5-10mg/kg; ATG-F 20mg/kg	Modified BUCY, n=118; Intensified BU-based MAC, n=259	CsA+MMF+short-term MTX	Real-time quantitative PCR	NR	67.4 ± 5.1%	1.06%	3-year OS 74.9 ± 2.4%; LFS 73.8 ± 4.8%; relapse rates 14.3 ± 4.0%; NRM 12.3 ± 3.5%	(16)
Jiafu Huang et al.	2020	China	75 patients aged over 50 years	AML (60); MDS (15)	BM+PBSC	*in vivo* TCD-haploSCT	r-ATG 7.5-10mg/kg	BUCY or BF or TBI+CY	CsA+MMF+short-term MTX	PCR	NR	64.00%	4.00%	2-year relapse 27.0% ± 5.6%; PFS 59.3% ± 5.8%; OS 63.0% ± 5.8%; GRFS 42.6% ± 5.9%	(17)
P Suo et al.	2020	China	27	MDS	BM+PBSC	*in vivo* TCD-haploSCT	r-ATG 2.5 mg/kg×4d	Modified BUCY	CsA+MMF+short-term MTX	Quantitative PCR	PET was given when a single CMV DNA > 1000 copies/mL or 600 copies/mL were observed twice.	59.30%	0	3-year DFS and 3-year OS 81.9%	(20)
P Ke et al.	2018	China	48	MDS	BM (9); PBSC (1); BM+PBSC (38); coinfusion of the cord blood	*in vivo* TCD-haploSCT	r-ATG 2.5 mg/kg×4d	Modified BUCY	CsA+MMF+short-term MTX	NR	NR	42%	0	2-year OS 64%; RFS 56%; relapse 12%; NRM 33%	(19)
L Gao et al.	2015	China	47	Ph+ ALL	BM+PBSC	*in vivo* TCD-haploSCT	r-ATG 2.5 mg/kg×4d	TBI+Ara-C+CY	CsA+MMF+short-term MTX	NR	NR	38.30%	8.51%	2-year OS 63.8%; LFS 59.5%	(18)
H Zhao et al.	2020	China	55	ALL	BM+PBSC or PBSC	*in vivo* TCD-haploSCT	NR	BUCY+TBI or nonmyeloablative regimens	NR	NR	NR	56.10%	NR	2-year LFS 65.6%; OS 77.0%	(21)
L Gao et al.	2017	China	174	AML (73); ALL (61); CML (22); MDS (18)	BM+PBSC	*in vivo* TCD-haploSCT	ATG-F 5mg/kg×4d	CCNU+BU+CY+Ara-C (AML,CML and MDS) CY+TBI+Ara-C (ALL)	CsA/Tacro+MMF+short-term MTX	PCR	NR	39.5% (Short-term Tacro); 37.5% (CsA)	NR	2-year OS 59.3% (Short-term Tacro), 55.7% (CsA); 2-year DFS 65.1% (Short-term Tacro), 61.4% (CsA)	(37)
Y Wang et al.	2014	China	224	AML (106); ALL (91); CML (14); MDS (13)	BM+PBSC	*in vivo* TCD-haploSCT	r-ATG 1.5 mg/kg×4d, n=112; r-ATG 2.5 mg/kg×4d, n=112	Modified BUCY, n=218; TBI based regimen, n=6	CsA+MMF+short-term MTX	Real-time Taqman CMV DNA PCR	>600 copies/mL	1-year 75.0% (ATG-6) and 78.6% (ATG-10)	0.89% (ATG-6) and 5.36% (ATG-10)	1-year relapse 7.6% (ATG-6), 4.6% (ATG-10); NRM 8.1% (ATG-6), 10.3% (ATG-10); OS 88.4% (ATG-6), 87.0% (ATG-10); DFS 84.3% (ATG-6); 86.0% (ATG-10)	(31)
S Kako et al.	2017	Japan	12	AML (5); ALL (1); CMML (1); Ph+ ALL (2); NHL (1); LCS (1); PMF (1)	PBSC	*in vivo* TCD-haploSCT	r-ATG 2.5 mg/kg×2d (-4 to -3)	BU+Mel, n=2; CY+TBI, n=6; Flu+Mel+TBI, n=3; Flu+BU+TBI, n=1	CsA+short-term MTX	NR	NR	41.67%	0	1-year OS 33.3%, PFS 24.3%, RR 59.0%, and NRM 16.7%	(34)
GJ Min et al.	2020	Korea	186	AML	BM or PBSC	*in vivo* TCD-haploSCT	r-ATG 1.25 mg/kg×4d	Flu+BU+TBI	CsA+short-term MTX	Real-time quantitative-PCR	NR	72.70%	19.40%	OS 52.3% (mismatched) and 55.3% (matched); GRFS 40.6% (mismatched) and 42.2% (matched); Relapse 22.5% (mismatched) and 8.6% (matched); NRM 28.9% (mismatched) and 27.1% (matched)	(35)
L Zhu et al.	2015	China	25	AML (7); ALL (17); Bi-lineage AL (1)	BM+PBSC+MSC (21) or BM+MSC (4)	*in vivo* TCD-haploSCT	r-ATG 2.5 mg/kg×4d (-4 to -1)	BU+Ara-C+CY	CsA+MMF+short-term MTX	NR	NR	92%	NR	14-month OS 53.28%	(28)
J Xu et al.	2020	China	72	T-ALL	BM or PBSC or BM+PBSC combined with CB	*in vivo* TCD-haploSCT	r-ATG 2.5 mg/kg×4d (-4 to -1)	Modified BUCY	CsA+MMF+short-term MTX	PCR	NR	19.40%	NR	3-year OS (66.6 ± 6.2)%; RFS (62.0 ± 6.5)%; relapse (24.2 ± 6.4)%; NRM (16.9 ± 5.1)%	(25)
J Wang et al.	2019	China	139	AML (100); ALL (39)	BM+PBSC or BM+PBSC+UCB	*in vivo* TCD-haploSCT	ATG-F 5 mg/kg×4d	BUCY+Me-CCNU+FLAG/CLAG, n=96; TBI+CY+Me-CCNU+FLAG/CLAG, n=43	CsA+MMF+short-term MTX	Real-time PCR	NR	100-day 59.8% (Cord-HaploSCT) and 47.6% (HaploSCT)	2.88%	2-year relapse 25.9% (Cord-HaploSCT) and 53.2% (HaploSCT); NRM 38.8% (Cord-HaploSCT) and 24.6% (HaploSCT); OS 35.5% (Cord-HaploSCT) and 22.7% (HaploSCT); PFS 35.5% (Cord-HaploSCT) and 17.9% (HaploSCT)	(26)
XN Gao et al.	2020	China	110	AML (58); MDS (6); CML (4); MDS/MPN (1); ALL (38), NHL (3), PCL (1)	PBSC	*in vivo* TCD-haploSCT	r-ATG 2.5 mg/kg×4d	Modified BUCY, n=95; TBI+CY, n=3; Flu+BU, n=4; BU+FLAG, n=8	CsA+MMF+short-term MTX	Real-time quantitative PCR	CMV DNA loads exceeded 1000 copies/mL	1-year 55.0%	1-year 7.9%	3-year NRM 30.5% (CMV DNAemia+) and 13.7% (CMV DNAemia-); 3-year OS 55.0% (CMV DNAemia+) and 60.4% (CMV DNAemia-)	(29)
HH Li et al.	2017	China	94	AML (46); Therapy-related AML (6); MDS transformed AML (5); MDS-refractory anemia with excess blast (1); ALL (26); CML (5); Lymphoma (5)	PBSC	*in vivo* TCD-haploSCT	r-ATG 2.5 mg/kg×4d	Modified BUCY, n=60; TBI+CY, n=28; BF, n=6	CsA+MMF+short-term MTX	NR	NR	1-year 62.1%	1-year 8.1%	3-year NRM 24.0% (HaploSCT) and 10.2% (MSD); relapse 39.0% (HaploSCT) and 22.6% (MSD); DFS 45.7% (HaploSCT) and 78.9% (MSD)	(30)
E Shmueli et al.	2014	Israel	102	Congenital disease; SAA; hematological malignancy; solid tumor	NR	*in vivo* TCD-haploSCT	ATG*	Flu+TT+TBI	NR	Real-time PCR	Higher than 50 copies/mL	66.70%	11.6%	The high rate of drug resistance as interlinked with severe disease in haplo-HSCT recipients.	(8)
SS Park et al.	2021	Korea	46	SAA	PBSC	*in vivo* TCD-haploSCT	r-ATG 5-10 mg/kg	TBI+Flu	Tacro+short-term course MTX	NR	NR	45.70%	NR	3-year OS 84.4%; 3-year TRM 11.2%	(36)
A Bertaina et al.	2014	Italy	23	SCID (8); SAA (4); FA (4); IPEX (1); CAMT (1); SDS (1); UNC13D-mutated HLH (1); DOCK-8-mutated HIEs (1); Osteopetrosis (1); Thalassemia (1)	PBSC	*ex vivo* TCD-haploSCT (αβ+ T and CD19+ B cells depletion)	r-ATG 4 mg/Kg×3d (-5 to -3)	BU+TT+Flu, n=3; Treo+TT+Flu, n=4; Treo+Flu, n=8; Flu+CY ± TBI, n=8	No posttransplantation pharmacologic GVHD prophylaxis	NR	NR	38% (CMV and adenovirus)	4.35%	The 2-year probability of both OS and DFS was 91.1%	(42)
AE Hammerstrom et al.	2018	USA	86	Leukemia (75); Lymphoma (8); MM (1); AA (2)	BM (83); PBSC (3)	PTCy-haploSCT	No	Mel+TT+Flu	MMF+Tacro	pp65 CMV antigenemia assay or PCR.	CMV antigenemia with ≥1 cell/million or detectable CMV DNA	Traditional 81%; Hybrid 53%; Intermediatedose 71%	8% (Traditional), 0% (Hybrid), and 0% (Intermediate dose)	100-day NRM 0 (Traditional), 13% (Hybrid), and 13% (Intermediate dose); 100-day OS 100% (Traditional), 80% (Hybrid), and 87% (Intermediate dose)	(60)
R Mitchell et al.	2019	Australia	19	Primary immunodeficiency disease; HLH; FA; AML; ALL	PBSC; BM	*ex vivo* TCD-haploSCT (αβ+ T and CD19+ B cells depletion)	ATG*	Treo+Flu+TT; Bu+Flu+TT; Treo+Flu; Bu+CY+Flu; Flu+CY; Flu+Mel+TT; TBI+Flu+Mel+TT	MMF (n=11) or CsA (=3) or combination CsA/MMF (n=5), or no prophylaxis (n=1)	CMV PCR screening	NR	50.00%	5.26%	100-day TRM 0% and 1-year TRM 15%; 5-years OS 80%	(43)
SH Kang et al.	2021	Korea	81	Malignant disease (45); Nonmalignant disease (36)	PBSC	*ex vivo* TCD-haploSCT (αβ T lymphocyte depletion)	Malignant disease r-ATG (2 mg/kg at -8d and 1 mg/kg at -7d); Nonmalignant disease r-ATG (2.5 mg/kg/day, -8d to -6d)	Flu+CY+TBI	NR	Quantitative real-time PCR	>2.49 log copies/mL	50.8% (GCV/FCV 44.4% *vs* GCV 62.6%)	15.4%; no significant difference in the incidence of CMV disease according to prophylaxis method	Interim-FCV prophylaxis effectively prevented CMV reactivation in those undergoing αβ T cell-depleted haploSCT.	(41)
I Airoldi et al.	2015	USA	27	ALL (9); AML (6); SCID (4); FA (3); Hyper-IgE syndrome (1); Refractory cytopenia of childhood (2); Kostmann syndrome (1); Osteopetrosis (1); SDS (1)	PBSC	*ex vivo* TCD-haploSCT (TCR-αβ+/CD19+ lymphocytes depletion)	No	TBI+TT+Mel; TBI+TT+CY; TBI+TT+Flu; Treo+TT+Mel; BU+TT+Flu; BU+CY+Mel; Treo+TT+Flu; Treo+Flu; TBI+CY+Flu; BU+Flu	No posttransplantation pharmacologic GVHD prophylaxis	NR	NR	55.50%	NR	81.5% survived at last follow-up	(44)
L Kaynar et al.	2017	Turkey	34	AML (24); ALL (10)	PBSC	*ex vivo* TCD-haploSCT (TcRαβ-depletion)	ATG-F 30 mg/kg (-12 to -9)	Flu+TT+Mel	MMF	PCR	NR	73.5% (AML 66.7%; ALL 90.0%)	0	1-year DFS 42%; OS 54%	(39)
HF Nazir et al.	2020	Oman	12	FHLH	PBSC	*ex vivo* TCD-haploSCT (CD3/CD19 depletion)	ATG-F 10 mg/kg (-6 to -3)	Treo+TT+Flu+Rituximab	CsA or Tacro or No pharmcologic prophylaxis	PCR	CMV viral load exceeded 500 copies/mL	75.00%	16.67%	3-year DFS 58.3%	(45)
F Erbey et al.	2018	Turkey	21	ALL (14); AML (7)	PBSC	*ex vivo* TCD-haploSCT (TcRαβ-depletion)	r-ATG 20mg/kg (-13 to -9)	Flu+TT+Mel	MMF with or without CsA	PCR screening	NR	81.00%	NR	5-year OS 71.1%; RFS 86.9%; TRM 16.3%	(40)
S Gaballa et al.	2016	USA	50	AML (27); MDS or MPD (3); ALL (14); NHL (5); AA (1)	DLI + CD34-selected stem cell	PTCy-haploSCT	No	TBI (12 Gy over 4 day)	Tacro+MMF	PCR	NR	100-day 67%	0	3-year OS 70%; PFS 68%; NRM 10%	(38)
R Crocchiolo et al.	2015	Italy	70	HL (35); NHL (20); MM (2); AL (11); CLL (2)	BM (66); PBSC (4)	PTCy-haploSCT	No	NMA, n=48; RIC, n=16; MAC, n=6	Tacro/CsA+MMF	PCR	Threshold of CMV viremia for PET was 3300 copies/mL	54.00%	4.29%	2-year OS 48%, TRM 26%	(53)
J Gaziev et al.	2018	USA	54	Thalassemia (45); Sickle cell anemia (7); HbS-b thalassemia (2)	PBSC and/or BM	*ex vivo* TCD-haploSCT (CD34 selection of PBSCs and BM, n=32; CD34 selection of PBSCs and CD3/CD19 depletion of BM, n = 8; TCRαβ/CD19 depletion of PBSCs, n = 14)	r-ATG 12.5 mg/kg over 4 days, n=6; ATG-F 50 to 25 mg/kg over 5 days, n=48	BUTT10CY200 preceded by HuAzFlu or BUTT10CY200 preceded by Flu with/without Rituximab prophylaxis	CsA +methylprednisolone or CsA+MMF	reverse-transcription PCR	NR	64.00%	0	OS 78% (TCR group) and 84% (CD34 group); DFS 69% (TCR group) and 39% (CD34 group)	(47)
L Prezioso et al.	2019	Italy	59	AML (32); ALL (6); NHL (6); HL (8); MF (4); MDS (2); MM (1); PCL (1)	PBSC (24); CD34+ (35)	*ex vivo* TCD-haploSCT (αβTCR/CD19+ depletion or selection of the CD34+ cells)	r-ATG 1.5 mg/kg ×4d (-9 to -6)	Flu+TT	No posttransplantation pharmacologic GVHD prophylaxis	PCR	NR	7.27%	1.69%	2-year OS 50.8%	(46)
D Huntley et al.	2020	Spain	118	AL (43); CL (9); Lymphoma (26) MDS/MM/Myelofibrosis (25); Other (15)	PBSC (110); BM (8)	PTCy-haploSCT	Only one patient received ATG	MAC,n=35; RIC,n=83	CsA or Tacro	RealTime CMV PCR	31 IU/ml or 137 IU/ml at different centers	63.90%	4.50%	1-year OS 70.3%	(55)
LJ Arcuri et al.	2020	USA	87	SAA	BM (81); PBSC (3); BM+PBSC (3)	PTCy-haploSCT	12 patients received r-ATG	Flu+CY+TBI	CsA+MMF or Tacro+MMF	Positive antigenemia or PCR	NR	100-day 61%, 1-year 62%, 2-year 62%	NR	2-year OS 79%; 2-year EFS 70%	(62)
M Slade et al.	2017	USA	104	AML (70); ALL (11); MDS (11); Other (12)	PBSC	PTCy-haploSCT	NR	MAC, n=43; NMA, n=61	CsA+MMF or Tacro+MMF	PCR	>40 000 IU/mL	55.00%	15%	51% survived at last follow-up	(69)
E Katsanis et al.	2020	USA	17	AL,CML, NHL	BM	PTCy/BEN-haploSCT (9); PTCy-haploSCT (8)	No	TBI+Flu or BU+Flu+Mel	MMF+Tacro	PCR	NR	12.5% in PTCy-BEN with 71.4% in PTCy	NR	2-year OS 83.3% in PTCy-BEN with 58.3% in PTCy	(64)
GC Irene et al.	2021	Spain	40	AL/MDS (28); MPN (1); Lymphoid malignancies (9); Others (2)	PBSC or BM	PTCy-haploSCT	No	RIC,n=1;MAC,n=39;	Tacro	Quantitative PCR	PET: a level of DNAemia of >1000 IU/ml in one blood sample or two consecutive samples with a level of >500 IU/mL	18-month 61%	2.50%	18-month OS 71.3%; PFS 67.4% with no differences by donor type	(59)
RV Raj et al.	2016	USA	43	AML/MDS (27); ALL (5); Myeloma (4); NHL/HL (4); Others (3)	BM (22); PBSC (21)	PTCy-haploSCT	No	Flu+CY+TBI, n=23; Flu+Bu+CY, n=15; Flu+Mel+TBI, n=5	Tacro+MMF	Quantitative nucleic acid amplified tests (NAAT)	NR	RIC with 40% in MAC	0 (RIC) and 7% (MAC)	NR	(63)
SR Goldsmith et al.	2016	USA	138	AML (93); MDS (15); Other (30)	PBSC	PTCy-haploSCT	No	MAC, n=58; RIC, n=80	Tacro+MMF or other	Real-time qPCR	NR	58.00%	16.67%	Post-transplant CMV viremia was not associated with a statistical difference in overall survival	(65)
J Montoro et al.	2020	Spain	42	AL (15); MM (5); Lymphoproliferative disorders (13); MDS (5); MPD (4)	BM (5); PBSC (37)	PTCy-haploSCT	No	TBF-MAC, n=9; TBF-RIC, n=2; BU+Flu+CY, n=11	MMF+Sirolimus	Quantitative real-time PCR assays	NR	52.00%	2.38%	1-year NRM 14%; EFS 75%; OS 82%; GRFS 47%. A higher cumulative incidence of CMV DNAemia requiring pre-emptive antiviral therapy in the haploidentical cohort.	(70)
N Cieri et al.	2015	Italy	40	AML (22); ALL (5); MDS (1); CML (1); HL (6); NHL (5)	PBSC	PTCy-haploSCT	No	Flu+Treo+Mel	MMF+Sirolimus	Quantitative PCR	PET was started when CMV DNA copy number was more than 1000 copies/mL or increased more than.5 log in peripheral blood plasma.	63.00%	15%	1-year OS 56%; DFS 48%	(71)
N Stocker et al.	2020	France	19	AML (10); MPN (1); MDS (1); ALL (4); NHL (3)	PBSC	PTCy-haploSCT	2.5 mg/kg, n=3; 5 mg/kg, n=16	RTC, n=13; TT+etoposide+CY+RIC, n=6	CsA+MMF	Quantitative PCR	PET was initiated when CMV was above 1000 IU/mL	46.00%	NR	2-year Relapse 19% (Control group) and 19% (PTCy group); PFS 73% (Control group) and 70% (PTCy group); OS 78% (Control group) and 79% (PTCy group)	(67)
Crocchiolo R et al.	2016	Italy and France	207	AL (44); HL (54); NHL (61); MM (13); MDS/MPS (25); Drepanocytosis (1)	PBSC (111); BM (96)	PTCy-haploSCT	NR	NMA/RIC, n=181; MAC, n=26	NR	NR	NR	42.00%	1.45%	Two-year OS 62% (Cohort 1); 65% (Cohort 2); 50% (Cohort 3); 42% (Cohort 4)	(56)
SR Goldsmith et al.	2021	USA	757	AML/ALL/MDS	BM or PBSC	PTCy-haploSCT	No	MAC or RIC/NMA	Tacro or CsA	PCR	NR	180-day 42%	100-day 2.8%	2-year mortaligy 49.5%	(51)
Y Lu et al.	2018	China	41	SAA (28)/VSAA (13)	BM+PBSC	*in vivo* TCD-haploSCT	ATG-r 7.5 mg/kg, n=42; ATG-F 20 mg/kg, n=47	BU+Flu+CY	Tacro+MMF+short-term MTX	PCR	Higher than 500 copies/mL in plasma	65.90%	4.88%	3-year OS 80.3% ± 5.1%; 3-year FFS 76.4% ± 5.1%	(13)
W-R Huang et al.	2016	China	130	AML; ALL; CML; Lymphoma	PBSC	*in vivo* TCD-haploSCT	r-ATG 2.5 mg/kg/day -5d to -2d	Modified BUCY, n=90; Modified BF, n=32; TBI+CY, n=8	CsA+MMF+short-term MTX	PCR	NR	1-year 61.0 ± 5.3%	1-year 8.0% ± 2.9%	3-year OS 45.6% ± 5.6%; LFS 44.2% ± 5.9%	(23)
BM Triplett et al.	2015	USA	17	ALL (6); AML (9); MLL (1); MDS (1)	PBSC	*ex vivo* T-cell depletion (CD45RA-depletion)	No	TLI+Flu+CY+TT+Mel	Sirolimus or MMF	PCR	NR	17.65%	0	76.5% survived at a median of 223 days	(49)
BM Triplett et al.	2018	USA	67	ALL (28); AML (22); MLL (4); MDS (8); Lymphoma (3); CML (2)	PBSC	*ex vivo* T-cell depletion (CD3-depletion,n=41; CD45RA-depletion,n=26)	No	CD3-depleted: Flu+TT+Mel+OKT3 (n = 21) or alemtuzumab (n=20)+Rituximab CD45RA-depleted: Flu+TT+Mel+lymphoid irradiation+CY	a short (<60 days) course of MMF	Quantitative PCR	NR	CD3-depleted 56%, CD45RA-depleted 19%	NR	180-day mortality CD3dep recipients 22% *vs* CD45RAdep recipients 15.4%	(50)
A Fayard et al.	2019	France	381	AL/MDS (208); HL/NHL (115); MPN (31); MM/solitary plasmacytoma (15); chronic leukemia (10); bone marrow failure syndrome (2)	BM (103); PBSC (278)	PTCy-haploSCT	No	RIC, n=307; MAC, n=73	an anticalcineurin +MMF	A single pp65 antigen-positive leukocyte or a positive viremia in peripheral blood	NR	48.80%	4.50%	Median of PFS 19.9 months; Median of OS 33.5 months	(52)
A Esquirol et al.	2021	Spain	236	AML (76); MDS (39); ALL (22); NHL (39); HL (31); CLL (8); CML/MPN (12); MM (5); biphenotypic acute leukemia (2); aplasia (1); prolymphocytic leukemia (1)	BM (45); PBSC (191)	PTCy-haploSCT	NR	Flu+BU; Flu+Bu+CY; TBF; Other	CsA+MMF or Tacro alone	PCR	>1000 IU/mL	69.00%	2.12%	12-month OS 64%; 12-month PFS 57%	(54)
Monzr M. Al Malki et al.	2017	USA	119	Acute leukemia (80); bone marrow failure (15); lymphoma (11); chronic leukemia (6); hemoglobinopathies (5); MM (2)	PBSC (81); BM (38)	PTCy-haploSCT	NR	MAC, n=46; RIC/NMA, n=73	Tacro/MMF	PCR	NR	100-day 69.2%	0	CMV reactivation was not associated with OS, RFS, relapse incidence, or NRM.	(57)
D Huntley et al.	2020	Spain	71	Acute leukemia (24); Chronic leukemia (6); Lymphoma (15); Myelofibrosis/MDS (18); Other (5)	PBSC (65); BM (6)	PTCy-haploSCT	No	MAC, n=17; RIC, n=54	Tacro-based, n=41; MMF-based, n=15	Real-time PCR	Higher than 600 IU/ml or higher than IU/ml at different centers	59.70%	4.23%	PTCy-haploSCT recipients may reconstitute CMV-specific T-cell immunity to the same extent as patients undergoing HLA-matched allo-HSCT	(58)
R Uppuluri et al.	2019	India	16	Primary immune deficiency disorder	BM (6); PBSC (10)	PTCy-haploSCT	NR	Flu+Mel, n=5; Flu+Treo, n=3; Treo+Flu+TBI, n=3; Treo+Flu, n=1; Flu+Treo+TBI, n=4	NR	NR	NR	43.70%	6.25%	Overall mortality 37.5%; OS 62.5%; Cytokine release syndrome (CRS) 75%	(61)
SR Solomon et al.	2015	USA	30	AML (16); ALL (6); CML (5); MDS (1); NHL (2)	PBSC	PTCy-haploSCT	No	Flu+TBI	Tacro+MMF	Quantitative CMV PCR	PET was initiated if viral reactivation was detected (higher than 400 copies/mL)	58.00%	0	2-year OS 78%; 2-year DFS 73%	(66)
C Oltolini et al.	2020	Italy	145	Myeloid disorders (106); Lymphoid disorders (39)	PBSC	PTCy-haploSCT	No	MAC, n=110; RIC, n=35	sirolimus+MMF, n=141; CsA+MMF, n=3	PCR	PET was started when plasmatic CMVDNA higher than 1000 copies/mL or increased >0.5 log.	61% (68%, haploSCT)	13.79%	Relapse 44%	(68)
AD Law et al.	2018	Canada	50	AML (28); MDS (8); MPN (6); ALL (2); Lymphoma (5); BPDCN (1)	PBSC	PTCy-haploSCT	r-ATG 4.5 mg/kg	Flu+BU+TBI	CsA	NR	NR	74%	8%	1-year OS 48.1%; NRM 38.2%	(72)
MQ Salas et al.	2020	Canada	52	AML (29); MDS (8); MPN (5); ALL (3); Lymphoproliferative disease (6); BPDCN (1)	PBSC	PTCy-haploSCT	r-ATG 4.5 mg/kg	Flu+BU+TBI	CsA	Quantitative PCR	>200 copies/ml	58%	4%	1-year OS 58.8 (44–70.9)%; 1-year RFS 53.3 (38.8–65.8)%	(73)
J Tischer et al.	2015	Germany	55	AML (33); CML (2); ALL (7); SAA (1); NHL (14); CLL (2)	BM+PBSC	*ex vivo* T-cell depletion (cTCR/TCD: CD6-depleted G-CSF-mobilized peripheral blood stem cells); PTCy-haploSCT	cTCR/TCD: r-ATG 20 mg/kg for 5 days; TCR/PTCY: No ATG	RIC or MAC	CsA+MTX or Tacro+MMF or MMF	Quantitative real-time PCR	NR	cTCR/TCD 42.9%; TCR/PTCy 14.8%	7.14% (cTCR/TCD) and 0 (TCR/PTCy)	cTCR/TCD: 1-year OS 39%, RFS 38%; TCR/PTCY: 1-year OS 59%; RFS 55%	(78)

HaploSCT, haploidentical stem cell transplantation; AML, acute myeloid leukemia; ALL, acute lymphoblastic leukemia; CML, chronic myeloid leukemia; AL, acute leukemia; MDS, myelodysplastic syndromes; AA, aplastic anemia; SAA, severe aplastic anemia; VSAA, very severe aplastic anemia; Ph+, Philadelphia chromosome-positive; PNH, paroxysmal nocturnal hemoglobinuria; CMML, chronic myelomonocytic leukemia; MM, multiple myeloma; NHL, non-Hodgkin lymphoma; PCR, polymerase chain reaction; PET, preemptive therapy; LCS, Langerhans cell sarcoma; PMF, primary myelofibrosis; BPDCN, blastic plasmacytoid dendritic cell neoplasm; PCL, plasma cell leukemia; SCID, severe combined immunodeficiency; FA, Fanconi anemia; IPEX, immunodeficiency with polyendocrinopathy and enteropathy X-linked; CAMT, congenital amegakaryocytic thrombocytopenia; SDS, Shwachmann-Diamond syndrome; HLH, hemophagocytic lymphohistiocytosis; UNC13D-mutated HLH, UNC13D-mutated hemophagocytic lymphohistiocytosis; DOCK-8-mutated HIEs, DOCK-8–mutated hyper-IgE syndrome; FHLH, familial hemophagocytic lymphohistiocytosis; MPD, myeloproliferative disease; HL, Hodgkin lymphoma; CLL, chronic lymphocytic leukemia; CL, chronic leukemia; MPN, myeloproliferative neoplasm; MPS, myeloproliferative syndrome; MLL, mixed lineage leukemia; BM, bone marrow; PBSC, peripheral blood stem cells; HSCT, hematopoietic stem cell transplant; MSC, mesenchymal stem cell; CB, cord blood; UCB, umbilical cord blood; DLI, donor lymphocyte infusion; TCD, T-cell depletion; PTCy, posttransplant cyclophosphamide; ATG, anti-thymocyte globulin; ATG-F, ATG-Fresenius; r-ATG, ATG-Genzyme; BU, busulfan; CY, cyclophosphamide; BUCY, busulfan cyclophosphamide regimen; CCNU, lomustine; Me-CCNU, simustine; Ara-c, cytosine arabinoside; BF, busulfan fludarabine regimen; FLAG, fludarabine+ cytarabine + granulocyte colony-stimulating factor; CLAG, cladribine + cytarabine + granulocyte colony-stimulating factor; Flu, fludarabine; TT, thiotepa; Treo, treosulfan; Mel, melphalan; Az, azathioprine; Hu, hydroxyurea; TBI, total body irradiation; TBF, thiotepa busulfan fludarabine; MAC, myeloablative conditioning, NMA, non-myeloablative; RIC, reduced-intensity conditioning; RTC, reduced toxicity conditioning; TLI, total lymphoid irradiation; CMV, cytomegalovirus; CsA, cyclosporine A; Tacro, tacrolimus; MMF, mycophenolate mofetil; MTX, methotrexate; GCV, ganciclovir; FCV, foscarnet; TCR, T-cell-replete; HLA, human leukocyte antigen; CMVR, cytomegalovirus retinitis; RRM, relapse-related mortality; OS, overall survival; LFS, leukemia-free survival; NRM, non-relapse mortality; TRM, transplant-related mortality; GVHD, graft-versus-host disease; aGVHD, acute graft-versus-host disease; FFS, failure-free survival; GFFS, GVHD-free and relapse-free survival; GRFS, GVHD-free relapse-free survival; PFS, progression-free survival; EFS, event-free survival; DFS, disease-free survival; RFS, relapse-free survival; RR, relapse rate; MSD, matched sibling donor; NR, not reported.

*The dose of ATG is not mentioned in the paper.

Johanna Tischer et al. retrospectively compared the incidence of virus infections and outcome in two different haploSCT settings (78). The first approach was the combination of T cell repletion and T cell depletion (CD6 deletion) using ATG prior to haploSCT (cTCR/TCD group). The second was T cell repletion (TCR) using high-dose posttransplantation cyclophosphamide (TCR/PTCy group). CMV reactivation occurred more frequently in the cTCR/TCD group (57%) than in the TCR/PTCy group (31%). Furthermore, pre-emptive treatment of CMV reactivation was successful in the TCR/PTCy group, whereas CMV DNA became undetectable in only 50% of the cTCR/TCD group.

## CMV-Specific Immune Reconstitution and Its Association With CMV Reactivation After haploSCT

### CMV-Specific T Cell (CTL)

We previously investigated CMV-specific T cell (CMV-CTL) reconstitution post *in vivo* TCD-haploSCT (85–87). The CD8+ T cell number in transplant recipients was comparable to that of controls at day 60 after transplantation. The median number of CMV-CTLs and their subsets was equal to those of the controls from day 30 to day 360. In addition, haploSCT recipients had a high frequency of CMV-CTLs with strong proliferation capacities and interferon-γ production at one year after transplantation (86). CMV-specific T cells with the central memory CD45RO+CD62L+ cell phenotype were significantly expanded when CMV was reactivated early after transplantation (87). Ruri Kato et al. demonstrated that there were considerably lower maximum numbers of CMV-CTLs in the CMV antigenemia resolved group than in the persistent group (median, 22.15 *vs*. 50 cells/μl) (88). Nevertheless, CMV-CTLs reached a peak more quickly in the resolved group than in the persistent group (median, 21 *vs*. 78 days) (88).

M Noviello et al. retrospectively explored either CD34 selection or posttransplant sirolimus as GVHD prophylaxis for haploSCT recipients (89). At 30 days, 21.7% of patients had CMV-specific T cells higher than 1 sfc/μL measured by enzyme-linked immunosorbent spot (ELISPOT), whereas CMV viremia occurred in only one patient who received anti-CMV treatment. At 90 days, 29.0% of patients reached this threshold, and no patients experienced clinically relevant viremia. At 180 days, 52.9% of patients finally reached the threshold, and none of them experienced CMV viremia. They found the protective value of 1 CMV sfc/μL against CMV reactivation posttransplant (89).

Dixie Huntley et al. performed a multicenter observational study to monitor CMV-specific T cell kinetics in PTCy-haploSCT patients and compared it with HLA-matched transplantation (58). In their analysis, CMV DNAemia developed at a similar frequency with equal numbers of CMV-specific T-cell at most time points examined between PTCy-haploSCT and MRD/MUD recipients. CMV DNAemia did not affect CMV-specific CD8+ and CD4+ T-cell reconstitution by the end of the follow-up period (day +180) in either allo-HSCT modality. They claimed that PTCy-haploSCT recipients may restore CMV-specific T-cell immunity to the same extent as HLA-matched allo-HSCT patients (58). The same group also reported that CMV infection was related to high levels of CD27−CD28− T cells, which behave like Tregs (90). They found a suboptimal correlation between CMV-specific CD4+ or CD8+ T cells and Tregs in peripheral blood (PB), which was weaker in patients with CMV reactivation prior to immunological monitoring. This suggests that recovery of PB Tregs and that of CMV-specific T-cell subsets show distinct kinetics, particularly after CMV reactivation.

More recently, Jasper J. P. van Beek et al. conducted longitudinal analysis of high-dimensional T-cell immunophenotypes in 21 recipients of PTCy-haploSCT (91). CMV-specific T-cells were primed early after PTCy-haploSCT and initially showed a proliferating/activated phenotype, that was quickly replaced by a terminal effector phenotype, while uncontrolled viral replication associated with lower abundance of distinct CMV-specific CD4+ T-cell immunophenotypes, hinting at a possible role of these cells in CMV control. CMV-specific T-cell features were similar to those of the CMV-seropositive donor one year posttransplantation, implying reestablishment of physiological homeostasis.

### NK

NK cells similarly play an essential role in defense against infections and leukemia relapse after hapoSCT. Fengyan Jin et al. explored NK cell dynamics in 29 patients after haploSCT between August 2011 and November 2014 (92). IFNγ-producing NK cells expanded in 19 patients after CMV reactivation, and the percentages of IFNγ-producing NK cells in these patients greatly increased from day 60 to 180 after transplantation compared to those of their donors. The percentage of KIR-expressing NK cells and IFNγ-producing NKG2C+ NK cells was significantly higher in haploSCT recipients with CMV reactivation than in those without CMV reactivation. Moreover, CMV reactivation was associated with expansion of the CD56^bright^CD16^dim/−^DNAM1^+^ NK cell subset between days 30 and 90 after haploSCT (93). Patients with increased CD56^bright^CD16^dim/−^DNAM1^+^ NK cells also had a remarkably higher CMV viral load (93).

Letizia Muccio et al. reported that CMV reactivation boosted the arrival of mature NK cells in pediatric patients with hematological malignancies receiving HLA-haploSCT after removal of both αβT cells and CD19 B cells (94). A memory-like NK cell subset expressing NKG2C and CD57 progressively expanded in most children. NKG2C+CD57+ NK cells were detected by month 3 after allo-SCT and expanded until at least month 12. These cells characteristically expressed high levels of killer Ig-like receptors (KIRs) and leukocyte inhibitory receptor 1 (LIR-1) and low levels of Siglec-7, NKG2A and interleukin-18Rα. Additionally, they poorly secreted interferon-γ in response to interleukin-12 and interleukin-18. The compromised response to these cytokines as well as their highly differentiated profile may reflect their skewing toward immune control of human cytomegalovirus.

Xiang‐Yu Zhao et al. from Peking University previously found that donor-recipient KIR ligand matching decreased CMV reactivation and refractory CMV infection by day 100 post-transplantation (95). This indicates that donor-recipient KIR ligand matching might improve the NK cell licensing process and promote NK cell-mediated control of CMV reactivation. The same group then prospectively assessed NK cell reconstitution in patients undergoing matched sibling transplantation and haploSCT (96). CD107a was increasingly expressed in NK cells after versus before CMV reactivation at days 60, 100, and 180 after transplantation, but CMV reactivation did not impact the maturation process of NK cells after transplantation. In addition, KIR expression and NKp30 expression were lower on NK cells in patients with CMV reactivation than in those without CMV reactivation at day 30. The NK-to-T-cell (NK/T) ratio was persistently higher in patients with CMV reactivation than in those without CMV reactivation from 30 days to one year after haploSCT.

An emerging report from Elisa Zaghi et al. demonstrated impaired adaptive NK cells expanded after CMV reactivation in PTCy-haploSCT (97). By a longitudinal single-cell computational profiling of multiparametric flow cytometry, they found that CMV accelerates NK cell immune recovery with the expansion of CD158b1b2j^pos^/NKG2A^neg^/NKG2C^pos^/NKp30^lo^ NK cells. The number of this subset is associated with CMV reactivation, further increases in recipients with multiple viral reactivations and persists for months after the infection. The transcriptional characteristics of FACS-sorted CD158b1b2j^pos^ NK cells confirmed the capacity of CMV to deregulate NKG2C, NKG2A, and NKp30 gene expression, thus mediating the expansion of NK cells with adaptive traits. These results imply that the dysfunction/exhaustion of “adaptive” KIR^pos^ NK cells in patients with CMV reactivated is induced, at least partially, by the CMV-induced expression of checkpoint inhibitors.

### γδ T

Fifty pediatric patients undergoing αβ T cell-depleted haploSCT between August 2012 and December 2015 were analyzed (98). CMV reactivation developed in 19 transplantations at a median of 30 days (range, 13-318 days) after haploSCT. Higher γδ T cells were observed in patients without CMV reactivation than in patients with CMV reactivation at day 30 (197.8 ± 153.9 *vs* 53.9 ± 58.7). There was a significantly higher incidence of CMV reactivation in patients with a low percentage of γδ T cells at day 30 than in patients with a high percentage of γδ T cells (78.0 ± 15.3% *vs* 22.2 ± 13.9%). No difference in day 30 γδ T cells was found between patients with and without CMV disease.

Irma Airoldi et al. prospectively monitored the functional and phenotypic characteristics of γδ T cells up to 7 months after αβ+ T cells and CD19+ B cells depleted haploSCT in 27 children (44). They reported that γδ T cells are the foremost T-cell population in patients during the first weeks and are mainly derived from the graft content and expanded *in vivo* after transplantation. Central memory cells predominated very early after haploSCT for both the Vδ1 and the Vδ2 subsets. Vδ1 cells are specifically expanded in patients with CMV reactivation and are more cytotoxic than those of children without reactivation.

CMV-specific T-cell, NK cell, and γδ T-cell are vital to immune control of CMV infection post haploSCT, but it seems that γδ T-cell is more likely responsible for viral reactivation in the context of *ex vivo* TCD-haploSCT. Although NK cells and γδ T cells are the first lymphocytes that recover after transplantation, CMV-specific T cells are dominant in number in case of viral infection. The majority of studies state that impaired T-cell and NK-cell reconstitution and increased risk of CMV infection after haploSCT, so seeking factors influencing CMV-specific immune reconstitution and interventions to improve immune reconstitution is urgent at the moment. Although data from Dixie Huntley et al. supported similar incidence of CMV infection and restored CMV-specific T cells after PTCy-haploSCT compared to MRD/MUD transplantation (58), the scarce number of MUD and MRD recipients and more sirolimus used in PTCy-haploSCT group preclude any definitive conclusion and further studies are warranted to validate their findings.

## Cellular Immunotherapy of CMV Infection

Delayed CMV-specific immune reconstitution has been consistently associated with the development of CMV infection and CMV disease after allo-SCT. Accordingly, adoptive transfer of CMV-specific T cells has been employed to treat CMV infection. Several clinical trials and case reports have confirmed the safety and efficacy of this strategy for the prophylaxis and treatment of CMV infection after haploSCT. **Table 2** lists cellular approaches currently in clinical trials and serves as evidence that CMV-targeting immune-based interventions could provide a safe, novel treatment option while offering clinical benefit to CMV reactivated recipients after haploSCT.

**Table 2:** Ongoing clinical trials using cytomegalovirus-specific cellular immunotherapy for allo-SCT patients including haploidentical SCT (accessed on 5 Oct 2021, ClinicalTrials.gov).

Intervention	Patients	Enrollment	Phase	Duration	NCT number	Status
Donor-derived viral specific T-cells (VSTs)	Stem cell transplant recipients who have evidence of viral infection or reactivation	450	Phase 1/Phase 2	2014-2024	NCT02048332	Recruiting
HLA-matched VSTs	EBV, CMV, adenovirus, and BK infections post allogeneic SCT	47	Phase 1	2021-2024	NCT04013802	Recruiting
Multivirus (CMV, EBV, AdV)-specific T cells	Chemo-refractory viral infections after allo-HSCT	149	Phase 3	2019-2022	NCT04832607	Recruiting
Third party donor derived CMVpp65 specific T-cells	CMV Infection or persistent CMV viremia after allogeneic hematopoietic stem cell transplantation	41	Phase 2	2014-2022	NCT02136797	Recruiting
Adaptive NK cells infusion post transplantation	CMV infection in patients post haploidentical transplantation	30	Not Applicable	2020-2021	NCT04320303	Recruiting
CMV-specific T cells	Relapsing or therapy refractory CMV infection after allogeneic stem cell transplantation	20	Phase 2	2016-2022	NCT03067155	Recruiting
CMV cytotoxic T cells (CTLs) manufactured with the Miltenyi CliniMACS Prodigy Cytokine Capture System	Refractory cytomegalovirus (CMV) infection post allogeneic hematopoietic stem cell transplantation (AlloHSCT), with primary immunodeficiencies (PID) or post solid organ transplant	20	Phase 2	2018-2023	NCT03266640	Recruiting
Direct infusions of donor-derived virus-specific T-cells using the Cytokine Capture System	Recipients of hematopoietic stem cell transplantation with post-transplant viral infections	12	Phase 2	2014-2022	NCT02007356	Recruiting
Emergency access to CMV pp65/IE-1 specific cytotoxic T lymphocytes	Recipients of allogeneic stem cell transplants with persistent or therapy refractory Infections	20	Phase 1	2008-2014	NCT00769613	Active, not recruiting
Viral specific T-Lymphocytes by Cytokine Capture System (CCS)	Infection with adenovirus, cytomegalovirus or Epstein-Barr Virus after hematopoietic cell transplantation or solid organ transplantation and in patients with compromised immunity	25	Phase 1/Phase 2	2021-2028	NCT04364178	Recruiting
CMV specific adoptive t-cells	Opportunistic cytomegalovirus infection occurring after stem cell transplant	20	Early Phase 1	2016-2022	NCT02982902	Recruiting
Virus specific T-cell (VST) infusion	Enhancing T-cell reconstitution before or after hematopoietic stem cell transplantation	60	Phase 1/Phase 2	2018-2023	NCT03475212	Active, not recruiting
CMV-specific T-cells	CMV in pediatric and adult immunocompromised patients or recipients of allogeneic stem cell transplantation	20	Phase 1	2020-2026	NCT03798301	Recruiting
Allogeneic cytomegalovirus-specific cytotoxic T lymphocytes	CMV reactivation or infection in participants who have undergone stem cell transplant or solid organ transplant	10	Early Phase 1	2020-2021	NCT03665675	Recruiting
Adoptive cell immunotherapy	Prophylaxis of cytomegalovirus infection in haploidentical transplantation of hematopoietic progenitors	15	Phase 2	2021-2022	NCT04056533	Not yet recruiting
Adoptive transfer of selected cytomegalovirus-specific cytotoxic T lymphocytes (CMV-CTL)	Patients at risk of CMV Disease after allogeneic stem cell transplantation (SCT)	78	Phase 2	2009-2013	NCT00986557	Recruiting
Donor derived cytomegalovirus specific T lymphocytes	Treatment of cytomegalovirus infection after allogeneic hematopoietic stem cell transplantation	30	Phase 4	2016-2021	NCT03004261	Recruiting

### Therapeutical CMV-Specific T-Cell Approaches

Feuchtinger T et al. treated 18 patients after allo-SCT from HLA–mismatched/haploidentical or HLA–matched unrelated donors with polyclonal CMV-specific T cells (99). These T cells were generated by isolation of interferon-γ–producing cells after stimulation with pp65 antigen. Patients with refractory CMV disease or viremia received a mean of 21 × 10^3^/kg pp65-specific T cells. CMV infection was cleared, or viral burden was significantly decreased in 83% of these patients, even in patients with CMV encephalitis. Viral control was related to improved antiviral T-cell reconstitution and *in vivo* expansion of CMV-specific T cells in 12 of 16 evaluable cases without inducing GVHD or acute side effects.

In another CMV infection refractory cohort (100), 27 of 32 treated patients after haploSCT cleared CMV within four weeks after adoptive T-cell therapy without recurrence. After cellular transfer, CMV-specific T cells expanded *in vivo* with improved cytokine production and proliferation ability. In addition, the expression of programmed death-1 (PD-1) on CMV-specific T cells was reduced. In the early effective group, patients who cleared viremia within four weeks after T-cell infusion, CMV-specific CD8+ IFN-γ+ and CD4+ IFN-γ+ T cells were rapidly and massively expanded *in vivo*, whereas in the late effective group, there was no significant expansion of CMV-specific T cells. Xiang-Yu Zhao et al. further evaluated the safety and efficacy of donor-derived CMV-specific cytotoxic T cells (CTLs) as a first-line therapy for CMV infection after haploSCT (101). They observed that first-line therapy with CTLs significantly reduced the incidence of CMV infection with lower 1-year treatment-related mortality and better 1-year overall survival. Moreover, first-line therapy with CTLs promoted the recovery of CTLs in patients, which correlated with CMV clearance.

A case report described two patients with drug-resistant CMV encephalitis after haploSCT successfully received donor CMV-specific CTLs (102). In the first case, a 27-year-old male developed CMV encephalitis during ganciclovir maintenance treatment after haploSCT. After administering foscarnet and donor CMV-specific CTLs, CMV-DNA of his cerebrospinal fluid (CSF) was negative by RT-PCR, and the lesions on brain magnetic resonance imaging (MRI) were reduced. Another case, a 57-year-old female, also experienced CMV encephalitis during maintenance treatment with ganciclovir after haploSCT. After intrathecal treatment with donor CMV-specific CTLs, the CMV load of the CSF was reduced.

### Prophylactic DLI

Prophylactic and therapeutic DLI are administered to improve posttransplant immune restoration to reduce both infectious complications and disease relapse. Michael Maschan et al. investigated low-dose memory (CD45RA-depleted) donor lymphocyte infusion (mDLI) after αβ T-cell depleted HSCT (103–105). The incidence of CMV reactivation was 45-50% in the experimental mDLI arm and 54-55% in the control arm. The median duration of CMV viremia was 3 weeks (range, 1-9) in the prospective cohort and 4 weeks (range, 1-26) in the historical cohort (105). Memory DLI was associated with improved CMV-specific T-cell reconstitution in a subcohort of CMV IgG seropositive recipients. Analysis of a subcohort of CMV seropositive recipients indicated remarkably better CMV-specific T-cell reconstitution on day 30 in the experimental arm (104). Compared to that of the historical cohort, restoration of CMV-specific immunity at day 30 was significantly enhanced in the prospective cohort (40% versus 25%) (105). Luca Castagna et al. prospectively evaluated a CD45RA+ depleted DLI in terms of reducing viral infection early after PTCy-haploSCT (106). CMV reactivation occurred in 28% of patients. Although the majority of the patients received the planned three infusions, only one patient developed grade 2 acute GVHD, and two patients had moderate chronic GVHD.

### Therapeutic DLI

Park HJ et al. reported the successful treatment of refractory CMV colitis after PTCy-haploSCT using CD45RA+ depleted DLI (107). After failure of ganciclovir and foscarnet, granulocyte colony-stimulating factor-primed, CD45RA+ depleted DLI was administered to treat refractory CMV colitis. CMV pp65-specific CTLs were found in recipients four weeks after DLI. Meanwhile, diffuse wall thickening involving the entire colon was also normalized in the abdominal CT scan.

As manipulated DLI approaches are still not widely used due to high cost and intensive labor, unmanipulated donor lymphocytes (U-DLIs), if feasible by harvesting CTLs directly from the peripheral blood of seropositive donors, are used for refractory or relapsed patients with CMV infection. Researchers from Turkey enrolled five pediatric patients receiving U-DLI for CMV infection after transplantation (108). Among them, three patients underwent haploSCT. One patient who was transplanted from an unrelated donor received U-DLI from his haploidentical mother. CMV titers were dramatically reduced after U-DLI in these patients.

## Summary and Outlook

Despite the use of prophylactic or preemptive treatments, CMV infection remains an obstacle for successful haploSCT and the improvement of immunologic reconstitution is the primary strategy for infection prevention. A higher rate of CMV reactivation occurred early after haploSCT compared to HLA-matched HSCT, but CMV disease rates were low after haploSCT, particularly in *in vivo* TCD-haploSCT and PTCy-haploSCT. It results from expansion of CMV-specific central memory T-cells in the setting of CMV antigenemia or acceptable CMV-specific T-cell reconstitution. Traditional *ex vivo* TCD-haploSCT successfully prevents lethal GVHD without any posttransplantation immunosuppression, but the small number of T cells in the graft results in impaired immune recovery, which could be overcome by novel *ex vivo* TCD-haploSCT and adoptive cellular therapy. *In vivo* TCD-haploSCT and PTCy-haploSCT indicated low treatment‐related mortality (TRM) and an acceptable safety profile, which appears to compare favorably with *ex vivo* TCD-haploSCT in terms of infections. However, synergistic immunosuppression by PTCy and ATG has led to a higher incidence of CMV infection. We now have a better understanding of CMV reactivation and immune reconstitution post haploSCT. Our data demonstrate that novel *ex vivo* TCD techniques followed by prophylactic and therapeutic DLI, a low dose of ATG, an intensified antiviral prophylaxis regimen, sirolimus-containing immunosuppressors and CMV-specific cellular immunotherapy can boost immune recovery and decrease the incidence of CMV reactivation. Furthermore, the majority of patients receiving the RIC regimen might be less susceptible to infections (63). In this context, it would be essential to perform a prospective study comparing the risk of infectious complications after *in vivo* TCD-haploSCT *vs. ex vivo* TCD-haploSCT or PTCy-haploSCT in patients who received a similar conditioning regimen.

CMV reactivation is associated with delayed immune reconstitution, although this reactivation could also leave a profound imprint on the recovering T cell compartment long-term following allo-SCT (91, 109, 110). Several studies have reported that CMV serostatus and CMV reactivation may be more predictive of T-cell restoration after allo-SCT than GVHD, highlighting the deep impact of this virus on reconstituting T-cells, considering the high incidence of CMV reactivation after haploSCT. More importantly, CMV infection is increasingly recognized as an immunomodulator in cancer patients (111), even in the context of allo-SCT, which is associated with a decreased risk of leukemia relapse, although it is still conflicting (112–115). There is evidence of a bidirectional relationship between CMV reactivation and acute GVHD (116, 117). We should take these into account and balance the merit and disadvantage of taking steps to enhance CMV-specific immune reconstitution and decrease CMV infection.

## Author Contributions

X-HL wrote the first draft of the manuscript, conducted the literature search, reviewed the abstracts, performed analysis and contributed to the final draft. YZ contributed to revising the manuscript and provided scientific input. Y-TC and L-PS conducted the literature search. LL revised and wrote the final draft, and contributed to the analysis. All authors contributed to the article and approved the submitted version.

## Funding

X-HL was supported by the National Natural Science Foundation of China [grant 81100388, grant 81470344].

## Conflict of Interest

The authors declare that the research was conducted in the absence of any commercial or financial relationships that could be construed as a potential conflict of interest.

## Publisher’s Note

All claims expressed in this article are solely those of the authors and do not necessarily represent those of their affiliated organizations, or those of the publisher, the editors and the reviewers. Any product that may be evaluated in this article, or claim that may be made by its manufacturer, is not guaranteed or endorsed by the publisher.
